# A substrate-independent framework to characterize reservoir computers

**DOI:** 10.1098/rspa.2018.0723

**Published:** 2019-06-19

**Authors:** Matthew Dale, Julian F. Miller, Susan Stepney, Martin A. Trefzer

**Affiliations:** 1 Department of Computer Science, University of York, York YO10 5DD, UK; 2 Department of Electronic Engineering, University of York, York YO10 5DD, UK; 3 York Cross-disciplinary Centre for Systems Analysis, University of York, York YO10 5DD, UK

**Keywords:** reservoir computing, physical computation, characterization

## Abstract

The reservoir computing (RC) framework states that any nonlinear, input-driven dynamical system (the *reservoir*) exhibiting properties such as a fading memory and input separability can be trained to perform computational tasks. This broad inclusion of systems has led to many new physical substrates for RC. Properties essential for reservoirs to compute are tuned through reconfiguration of the substrate, such as change in virtual topology or physical morphology. As a result, each substrate possesses a unique ‘quality’—obtained through reconfiguration—to realize different reservoirs for different tasks. Here we describe an experimental framework to characterize the quality of potentially *any* substrate for RC. Our framework reveals that a definition of quality is not only useful to compare substrates, but can help map the non-trivial relationship between properties and task performance. In the wider context, the framework offers a greater understanding as to what makes a dynamical system compute, helping improve the design of future substrates for RC.

## 1. Introduction

Reservoir computing (RC) first emerged as an alternative method for constructing and training recurrent neural networks [1,2]. The method primarily involved con- structing a random fixed recurrent network of neurons, and training only a single linear readout layer. It was found that random networks constructed with certain dynamical traits could produce state-of-the-art performance without the laborious process of training individual internal connections. The concept later expanded to encompass any high-dimensional, input-driven dynamical system that could operate within specific dynamical regimes, leading to an explosion in new RC substrates.^1^

The ability to perform useful information processing is an almost universal characteristic of dynamical systems, provided a fading memory and linearly independent internal variables are present [3]. However, each dynamical system will tend to suit different tasks, with only some performing well across a range of tasks.

In recent years, RC has been applied to a variety of physical systems such as optoelectronic and photonic [4,5], quantum [6–8], disordered and self-organizing [9,10], magnetic [11,12] and memristor-based [13] computing systems. The way in which each substrate realizes a reservoir computer varies. However, each tends to implement, physically or virtually, a network of coupled processing units.

In each implementation, the concept is to use and exploit the underlying physics of the substrate, to embrace intrinsic properties that can improve performance, efficiency and/or computational power. Each substrate is configured, controlled and tuned to perform a desired functionality, typically requiring the careful tuning of parameters in order to produce a working, or optimal, physical reservoir computer for ad hoc problems.

Despite the recent advances of new physical reservoir systems, basic questions for RC are still unanswered. These open problems are summarized and explained in [14]. Relevant questions include: What class of problems can RC solve efficiently? What is the role of heterogeneous structure in RC? What are the limits and benefits of a given physical system for RC? What are the benefits of a physical implementation? To answer these questions, and for the field to move forward, a greater understanding is required about the computational expressiveness of reservoirs and the substrates they are implemented on, if not to at least determine what tasks, for what substrates, are realistically solvable.

In the terminology used here, a *reservoir* represents the resulting abstract system and its dynamics instantiated by (typically, but not limited to) a single, typically static, configuration of the substrate. For an artificial recurrent neural network, implemented *in silico*, configuration may refer to a set of trained connection weights, defined neuron types and topology. For another substrate, configuration may refer to the physical morphology, physical state, external control signals or complexification of the driving input signal. The number of possible reservoir systems realizable by a substrate depends upon the number of free parameters, and the distinct dynamical behaviours resulting from those parameters. For unconstrained substrates, limited only by the laws of physics, this number may be vast. Yet this does not imply that every such configuration and corresponding reservoir is practical or useful. This also does not imply that each new configuration leads to a different reservoir system; the same or similar dynamical behaviour may be produced by different configurations. The mapping between substrate configuration and instantiated reservoir may be complex.

Here we present a practical framework to measure the computational expressiveness of physical or virtual substrates, providing a method to characterize and measure the RC *quality* of substrates.

A higher quality substrate is one that can realize more *distinct* reservoirs through configuration, giving it greater expressiveness and higher dynamical freedom, and so a greater *capacity* to tackle very different tasks. Quality is quantified and measured here as the number of distinct reservoirs, or dynamical behaviours, a single substrate can exhibit. The number of reservoirs, rather than configurations, is what is important. This does not imply that substrates with fewer available configuration degrees of freedom perform poorly at every task; they may perform very well at specific tasks within their dynamical range, but are likely to perform poorly when evaluated across a broad range of tasks.

To characterize the quality of different substrates, we present the CHARC (CHAracterization of Reservoir Computers) framework. The framework has a basic underlying structure, which can be extended if needed. To demonstrate the framework, it is applied to three different substrates: echo state networks (ESNs) [15], simulated delay-based reservoirs (DRs) [4,16] and a physical carbon nanotube (CNT) composite [9]. The definitions, techniques and substrate-independence of the framework are evaluated using a number of common benchmark tasks.

The rest of the paper describes the framework and the techniques used within it, beginning with a description of the workflow, the task-independent properties and search procedure used to characterize the substrate.

## 2. Framework outline

**Figure 1.**
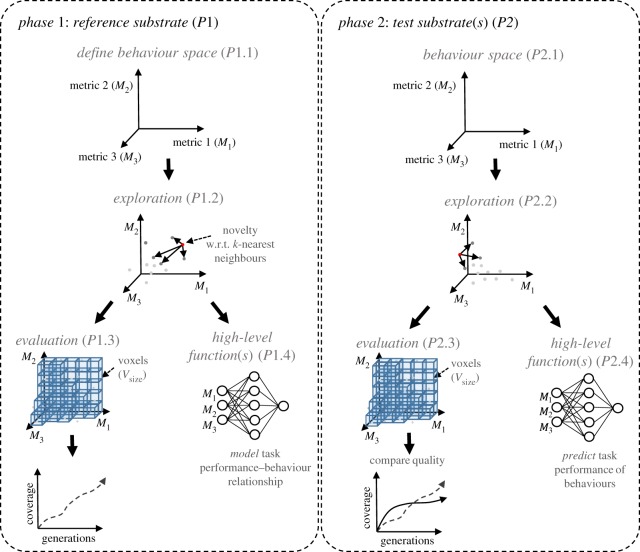
Framework phases and levels. (Online version in colour.)

The basic structure and flow of the framework is presented in figure 1. The complete characterization process is divided into a series of phases and levels. In phase one (*P*1), a reference substrate is evaluated, forming the basis against which to compare quality values. In phase two (*P*2), the test substrate is assessed and compared to the reference.

### (a) Basic levels

The three basic levels required for each phase are *definition*, *exploration* and *evaluation*. Additional levels may be added, providing further functions that can be used to manipulate, model and learn from the data produced by the characterization process. Here, an additional level is used to validate and determine the reliability and substrate-independence of the overall framework; others are also possible, see §2b.

In general, each level requires the results from the previous level. Techniques applied at each level are flexible, and may be substituted with alternative approaches. The techniques and measures used here are simple, and provide a good foundation to demonstrate the framework's concept.

**Figure 2.**
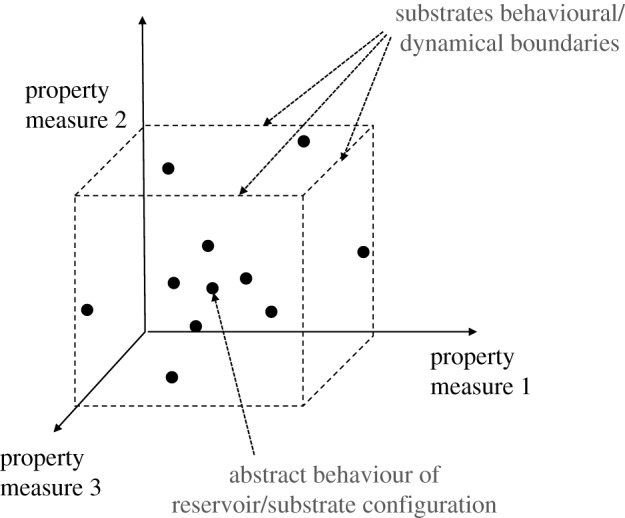
Example of a three-dimensional behaviour space. Here each abstract behaviour is relative to the three chosen property measures. Given enough time to explore the space, the substrate's dynamical/behavioural limitations become apparent.

The *definition* level (*P*1.1, *P*2.1) defines the reservoir *behaviour* space to be explored. The behaviour space represents the abstract behaviour of the configured substrate relative to measures of dynamical properties, and is the space in which *quality* is measured. The framework uses *n* measures (see example in figure 2) to define the axes of the *n*-dimensional behaviour space. See §2c for the measures used here.

The *exploration* level (*P*1.2, *P*2.2) measures the quality, by determining how much of the behaviour space is realizable through substrate configurations. An exhaustive search of the substrate's parameter space is infeasible. Instead, the use of diversity search algorithms [17] is recommended. These exploration techniques, based on evolutionary algorithms, can characterize the behaviour range and dynamical freedom of the substrate.

The *evaluation* level (*P*1.3, *P*2.3) estimates quality, by using the behaviours discovered from the exploration level. The behaviour space is divided into discrete voxels; the total number of voxels occupied by discovered behaviours provides the final quality value of the substrate. In *P*2.3, the quality of the test substrate is compared with the quality of the reference substrate from *P*1.3.

### (b) Additional levels providing further functions

Additional levels can be added to the framework to extract further features about the substrate and the behaviour space representation. They need not necessarily relate to the evaluation level (the quality value), and may work independently of it. Example additional levels include: modelling the relationships between the behaviour space and task-specific performances; modelling the relationships between the behaviour space and configuration space. Such relationships can be modelled and learnt using machine learning techniques.

Here, one additional level is created: a *learning* level (*P*1.4, *P*2.4). The learning level is used here to evaluate whether the framework is reliable (that the behaviour metrics capture the underlying reservoir properties) and substrate-independent (that behaviours learned in one substrate can be transferred to a different substrate). To achieve independence, the reliability of the behaviour space representation should be high. In reality, due to noise and translation between the simulated and physical domain, we require reliability above some acceptable threshold.

Further levels building on the exploration and learning levels are also possible. For example, the discovered behaviours can provide a reduced search space from which to rank and find optimal reservoirs for a particular task. As the number of tasks increases, this reduced search space decreases the required time to find good task-specific reservoirs without having to repeatedly search over the full configuration space.

### (c) Task-independent properties

In order to form a suitable behaviour space, we need to define each dimension of the space carefully. Some potentially interesting properties are difficult, if not impossible, to transfer across all substrates. For example, measures that require access to the system's internal workings will not transfer to black-box systems. Measures used with the framework must represent only the observable behaviour of the system, independent of its implementation.

In general, the behaviour space should be created using as many uncorrelated measures as possible, representing different computational and dynamical properties. This will improve the reliability of the framework, but result in a larger space to explore, requiring more evaluations to build a useful characterization.

In the work here, three common property measures are taken from the RC literature to form the behaviour space. These measures are the kernel rank (KR), generalization rank and memory capacity.

KR is a measure of the reservoir's ability to separate distinct input patterns [18]. It measures a reservoir's ability to produce a rich nonlinear representation of the input *u* and its history *u*(*t* − 1), *u*(*t* − 2), …. This is closely linked to the *linear separation property*, measuring how different input signals map onto different reservoir states. As many practical tasks are linearly inseparable, reservoirs typically require some nonlinear transformation of the input. KR is a measure of the complexity and diversity of these nonlinear operations performed by the reservoir.

GR is a measure of the reservoir's capability to generalize given similar input streams [18]. It attempts to quantify the generalization capability of a learning device in terms of its estimated VC-dimension [19], i.e. how well the learned nonlinear function generalizes to new inputs. In general, a *low* GR symbolizes a robust ability to map similar inputs to similar reservoir states, rather than overfitting noise.

Reservoirs in ordered dynamical regimes typically have low ranking values in both KR and GR, and in chaotic regimes have both high. A rule-of-thumb is that good reservoirs possess a high KR and a low GR [20]. In terms of matching reservoir dynamics to tasks, the precise balance will vary.

A unique trait that physical and unconventional substrates are likely to possess is the ability to feature multiple time-scales and possess tunable time scales through reconfiguration, unlike their more conventional reservoir counterparts.

Another important property for RC is memory, as reservoirs are typically configured to solve temporal problems. (A substrate without memory may still be computationally interesting for solving non-temporal problems.) A simple measure for reservoir memory is the *linear short-term memory capacity* (MC). This was first outlined in [21] to quantify the echo state property. For the echo state property to hold, the dynamics of the input-driven reservoir must asymptotically wash out any information resulting from initial conditions. This property therefore implies a fading memory exists, characterized by the short-term memory capacity.

A full understanding of a reservoir's MC, however, cannot be encapsulated through a linear memory measure alone, as a reservoir will possess some nonlinear memory. Other memory measures proposed in the literature quantify other aspects of memory, such as the quadratic and cross-memory capacities, and total memory of reservoirs using the Fisher memory curve [3,22]. The linear measure is used here to demonstrate the framework; additional measures can be added as needed.

### (d) Behaviour space exploration

To characterize the reservoir behaviour space, the search must explore without optimizing towards any particular property values. A balance between properties is essential to match reservoir dynamics to tasks. However, determining the right balance is challenging. During the characterization process, the exact balance required for specific tasks is irrelevant. Instead, the focus is to explore and map the space of possible trade-offs the substrate can exhibit, and use this to determine substrate quality.

For the framework to function, the mapped reservoir behaviour space requires substrate-independence, so the exploration cannot be conducted, or measured, in the substrate-specific parameter space. Also, the exploration must be able to function without prior knowledge of how to construct reservoirs far apart from each other in the behaviour space, as diversity in observed dynamics is not easily related to diversity in substrate-specific parameters.

Here, exploration is performed using the open-ended novelty search (NS) algorithm [23–25], one of several possible diversity algorithms [17]. NS increases the selection pressure of an underlying evolutionary algorithm towards novel behaviours far apart in the behaviour space. The full details of our NS implementation are given in appendix A.

## 3. Phase one: reference substrate

Phase one establishes a suitable *reference* substrate to compare against. Here, we use recurrent neural networks (RNNs) that closely resemble ESNs [21] as the reference. These are well established state-of-the-art reservoir ‘substrates’. RNNs are flexible, universal approximators of dynamical systems [26] producing a vast range of dynamics when reconfigured.

For a standard ESN, the reservoir state update equation *x*(*t*) is

3.1 $$x(t)=f(W_{\mathrm{in}}u(t)+Wx(t-1)+W_{\mathrm{fb}}y(t)),$$

where *f* is the neuron activation function (typically a sigmoid) and the weight matrices *W*_in_, *W* and *W*_fb_ are matrices of connection weights to inputs (*W*_in_), internal neurons (*W*) and from the output to internal neurons (*W*_fb_). In many cases, the feedback weights *W*_fb_ are unused and the other weight matrices are selected from a random distribution, then scaled globally.

The final trained output *y*(*t*) is given when the reservoir states *x*(*t*) are combined with the trained readout layer *W*_out_:

3.2 $$y(t)=W_{\mathrm{out}}x(t).$$

Training of the readout is typically carried out in a supervised way using one-shot linear regression with a teacher signal. A practical guide to creating and training ESNs is given in [27].

### (a) Demonstrating and validating the framework

In a typical use of the framework, one would now perform the various levels of phase one to characterize the ESN reference substrate. Here we do more, performing several experiments to demonstrate why certain choices have been made, to explore the framework in action and to determine the reliability of the results.

Here, four sizes of ESNs are used for the purpose of demonstrating the framework. The four network sizes chosen have 25, 50, 100 and 200 nodes. This small spectrum from simple to complex reservoirs provides a useful test suite. Each size is a constrained version of the general ESN substrate, and exhibits different ranges of dynamical behaviours.

### (b) Novelty versus random search

Here we apply the exploration process *P*1.2, and evaluate the use of NS by comparing it to random search, determining its usefulness for characterizing a substrate. If NS performs well, if it discovers a greater variation in behaviours than random search within the same time, across network sizes, we argue it will continue to be advantageous for different substrates.

First, we compare NS and random search visually. The hypothesis here is that NS can cover a greater volume of the behaviour space within the same number of search evaluations.

**Figure 3.**
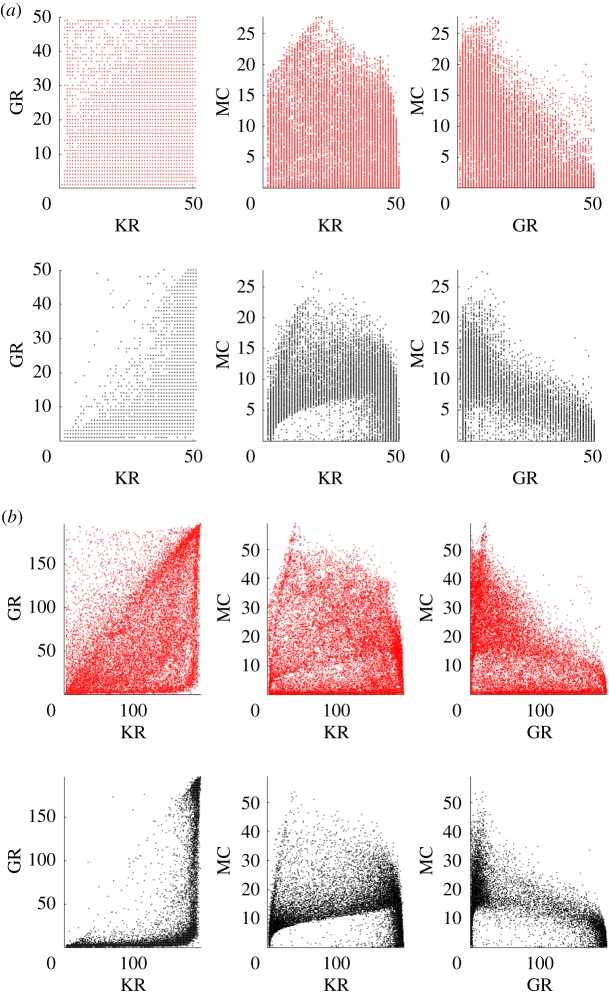
Behaviours discovered using NS (red, top row) and random search (black, bottom row) for network sizes of (*a*) 50 nodes, (*b*) 200 nodes. The three-dimensional behaviour space is shown as three projections. (Online version in colour.)

The results of this experiment show that for every network size, NS expands further in all dimensions of the behaviour space. In figure 3, the explored spaces of the 50 and 200 node ESNs using both search techniques are plotted. In total, approximately 20 000 configurations from 10 separate runs are displayed.

Random search (in black, bottom row), which selects weights and scaling parameters from uniform random distributions, appears to produce similar patterns in the behaviour space across all network sizes. These patterns show sparse regions that are difficult to discover, and dense areas that are frequently visited despite different configuration parameters. As network size increases, random search tends to find it more challenging to uniformly cover the behaviour space, suggesting it becomes less effective as substrate complexity increases.

NS (in red, top row) covers the behaviour space more uniformly, filling sparse regions and expanding into areas undiscovered by the random search. It does this within the same number of network evaluations, showing itself to be a more effective search technique than simply sampling the configuration space from a random uniform distribution.

### (c) Quality measure

Here we perform the evaluation process *P*1.3 on the behaviours discovered by NS above, in order to evaluate the voxel-based quality measure proposed to quantify the coverage of the behaviour space, and thus quality.

To measure quality and coverage of the behaviour space, standard statistical measures of dispersion such as standard deviation, mean absolute deviation and interquartile range are not suitable by themselves: they downplay outliers, whereas the aim is to measure both the volume and the boundaries of the region explored. For this reason, a voxel-based measure is adopted here. Discovered behaviour instances occupying the same voxel are counted once, thereby grouping similarly behaved reservoirs as a single behaviour voxel.

In our three-dimensional example, the discovered behaviours define the bounds of the measurable behaviour space: a large cube. The space is then discretized and partitioned into smaller voxel cubes. The smallest possible voxel size is 1 × 1 × 1: the smallest discretized value of the KR and GR property measures.

Voxel size needs to be chosen carefully in order to accurately compare substrates. If the voxel size is too small, every occupied voxel will contain exactly one explored reservoir behaviour, and the quality measure will merely record the number of search points evaluated. If the voxel size is too large, the quality measure will be too coarse grained to make distinctions.

Experiments to investigate the effect of voxel size are given in appendix E. These lead us to choose a voxel size of *V*_size_ = 10 × 10 × 10 for the rest of this paper.

The quality of a tested substrate is equal to the final coverage value. As voxel size and total number of evaluations both affect this value, the reference and test substrate should be compared using the same framework parameters.

### (d) Reliability of the behaviour space

In the last part of phase one addressed here, *P*1.4, the reliability of the behaviour space is measured, to demonstrate that the framework produces usable results. The outcome of this measure is also used to determine that the behaviour space is independent of the substrate implementation, *P*2.4, §4d. If the reliability is poor, independence is difficult to measure and interpret.

To assess reliability and independence, concepts such as the *representation* relation and commuting diagrams from Abstraction/Representation (A/R) theory [28] are adapted to form a testable hypothesis. In A/R theory, a framework is proposed to define when a physical system computes. Using those concepts, one can assess whether an abstract computational model reliably represents computation performed by a physical system.

Our hypothesis for the framework is that if the abstract reservoir space is truly representative of system dynamics, and independent of its implementation, it should hold that similar behaviours across substrates produce similar task performances. This hypothesis was conceived using A/R commuting diagrams as a template, where if the computational model faithfully represents the computation of the physical system, one can predict how the physical system states will evolve.

To test the hypothesis, first the relationship between task performance and reservoir behaviour is modelled. The reliability of this model, measured as the prediction error of the model, indicates how well the behaviour space captures the computation occurring within the substrate.

As explained in [14], relating property measures to expected performance across many tasks is a non-trivial problem, as good properties for one task are often detrimental to another. Therefore, no single set of measured values will lead to high performance across all tasks. However, the relationship between behaviour measure values and a single task are often simpler to determine; these are the relationships to be modelled.

To create the prediction model, four common RC benchmark tasks are selected: the nonlinear autoregressive moving average (NARMA) task with a 10th and a 30th order time-lag; the Santa Fe laser time-series prediction task; the nonlinear channel equalization (NCE) task. Each task requires a different set of reservoir properties. Full details of the tasks are provided in appendix B.

The modelling process of matching behaviours to task performances is framed as a regression problem. The model is created using standard feed-forward neural networks (FFNNs) and trained using a sample of the behaviours discovered in the exploration process, and their evaluated task performances. The inputs of the FFNNs are MC (continuous-valued), KR and GR (discrete values). The output of the FFNNs is the predicted task performance (continuous-valued) of each behaviour, measured as the normalized mean squared error (NMSE).

The prediction error of the FFNNs is measured on the test sample, as the root mean squared error (RMSE) between the predicted NMSE and the behaviour's actual evaluated NMSE for a given task. That is, the prediction error PE is

3.3 $$\mathrm{PE}={\left(\frac{1}{N}\sum_{i\in \mathrm{test}}{\left({\mathrm{ptp}}_{i}-{\mathrm{atp}}_{i}\right)}^{2}\right)}^{1/2},$$

where *N* is the size of the test set, ptp is the predicted task performance NMSE, and atp is the actual task performance NMSE.

In the experiment, multiple FFNNs of the same size are trained per task, and per substrate network size (see appendix F for experimental details). If the behaviour space provides a reliable representation, the mean prediction error of the trained FFNNs should be low, since reliability implies a strong relationship is present, that is not too difficult to model, and that is similar when network size changes.

Some difference in prediction error is present between models trained with different network sizes. This is due to different behavioural ranges, resulting in an increase or decrease in complexity of the modelled relationships. For example, reservoirs in the behaviour space around KR = GR = MC ≤ 25 tend to have similar poor performances for the NARMA-30 task because they do not meet a minimum requirement (MC≥30). This means the task is easier to model for small networks, as performance tends to be equally poor for all behaviours. Similarly, when larger ESNs are used to model the relationship, prediction error will likely increase as the distribution of errors increases and the complexity of the model increases. Patterns such as this are task-dependent, adding another level of complexity to the modelling process. For some tasks, to reliably model the relationship requires a greater variety of behaviours than smaller ESNs can provide. Therefore, FFNNs trained on the behaviour space of a 200 node network perform better than ones provided by the smaller networks, despite the apparent increase in complexity.

**Figure 4.**
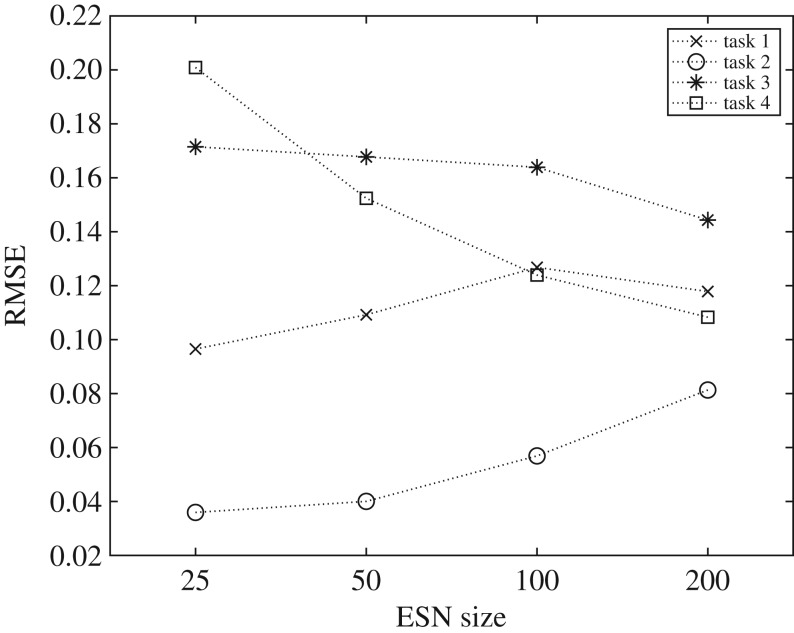
Mean prediction error (RMSE) of FFNNs across all tasks and ESN sizes. Task 1: NARMA-10, Task 2: NARMA-30, Task 3: Santa Fe laser, and Task 4: nonlinear channel equalization. (The spread in values across the 10 FFNNs evaluated at each point is too small to see on this plot.)

The mean prediction errors of the FFNNs, for each task and substrate size, are shown in figure 4. Overall, the prediction errors are low, with typical values of less than 0.16. Depending on the task, errors increase or decrease as substrate network size increases. The prediction error for task 3 (Santa Fe laser) and task 4 (nonlinear channel equalization) decreases with substrate size, suggesting the model improves when trained using a larger variety of behaviours. However, these two tasks are particularly challenging to model (with a typical RMSE > 0.1) because of outliers in the training data coming from poor (high task error) and very good (low task error) reservoirs, typically with an NMSE≪0.1.

For the NARMA tasks, task 1 (NARMA-10) and 2 (NARMA-30), the prediction error increases as the network size increases. Prediction accuracy of the model therefore tends to degrade when trained with larger behaviour spaces, in contrast with tasks 3 and 4. However, this increase in error happens as variation in task performances increases, mirroring the same modelling problem for tasks 3 and 4. The lowest task errors for the NARMA-10 drop from an NMSE ≈ 0.13 to an NMSE ≈ 0.01 as size increases. The same also occurs for the NARMA-30 task, with the lowest errors decreasing from an NMSE ≈ 0.48 to an NMSE ≈ 0.14.

From these results, a strong correlation emerges between the variance in task performance (NMSE) of each behaviour space and the prediction error (RMSE). This suggests refocusing the learning process: instead of trying to reliably model *all* behaviours, including the poor reservoirs, try to reliably model and predict only the best performing behaviours. The additional experiments in appendix F show the effect of this refocusing. The RMSE is significantly reduced when modelling behaviours below a task performance error threshold, rather than all behaviours. The results show the behaviour representation and model is most reliable when representing only the better task performing behaviours.

Overall, the results of this evaluation step suggest the behaviour space provides a sufficiently reliable representation of the substrate's computational capabilities. However, given that the provided behaviour measures are known not to capture all the interesting dynamics of the system, there is room to improve the behaviour representation and the modelling process.

## 4. Phase two: test substrates

Phase two of the framework, *P*2, evaluates the test substrate(s) against the phase one reference substrate(s). The behaviour space of the test substrate(s) is explored in level *P*2.2; the quality is determined relative to the reference in level *P*2.3; here the framework's substrate-independence is evaluated in level *P*2.4.

To demonstrate and evaluate the framework, two test substrates are characterized here: a simulated delay-based reservoir, and a physical CNT-based system. Each chosen substrate poses a unique challenge for the framework. These include differences in implementation (simulated or physical), structure (spatial or temporal) and levels of noise in each system.

### (a) Delay-based reservoir

The first substrate to be characterized is based on the delay-line reservoir (DR) system [4,16,29], using a single nonlinear node and a delay line. This particular reservoir system mimics the structure of a recurrent network of coupled processing nodes in the time domain rather than spatially. By applying time multiplexing and nonlinear mixing to the input signal, a virtual network of processing nodes is created. To date, DRs have produced excellent performances across different RC benchmarks [4,30,31].

Delay-feedback dynamical systems possess high-dimensional state spaces and tunable memory making them ideal candidates for RC. The dynamics of these systems are typically modelled using delay differential equations of the type:

4.1 $$\frac{d}{dt}x(t)=-x(t)+f(x(t-\tau ),J(t)),$$

where *t* is time, *τ* is the delay time, *f* is the nonlinear function and *J*(*t*) is the weighted and time multiplexed input signal *u*(*t*).

The DR technique is popular for optical and optoelectronic dynamical systems as it enables the exploitation of properties unique to these systems. It also provides a simple structure to overcome technical hardware challenges. These include exploiting high bandwidth and ultra-high speeds, and removing the demanding requirement of large complex physical networks. The technique however is not limited to these systems. It also offers a novel approach to implement networks efficiently on other hardware platforms. This is particularly useful when few inputs and outputs are available, creating the required state and network complexity in the time-domain to solve tasks. Examples include electronic circuits [4,32], Boolean nodes on a field-programmable gate array (FPGA) [33], a nonlinear mechanical oscillator [34] and spin-torque nano-oscillators [8]. However, the DR technique also has potential shortcomings including a serialized input, pre-processing required on the input and limits determined by the length of the delay line. To overcome some of these shortcomings, more complex architectures of multiple time-delay nodes have been proposed, leading to improved performances compared to single-node architectures [35].

The DR system characterized here consists of a simulated Mackey–Glass oscillator and a delay line, inspired by Appeltant *et al*. [4]. This same system was also realized physically using an analogue electronic circuit in [4]. Details on the implementation of the Mackey–Glass system and the time-multiplexing procedure are provided in appendix Cc.

### (b) Physical carbon nanotube-based reservoir

The second substrate to be characterized is a physical material deposited on a micro-electrode array. The substrate is electrically stimulated and observed using voltage signals and configured through the selection of input and output locations on the array. The material is a mixed CNT–polymer composite, forming random networks of semi-conducting nanotubes suspended in a insulating polymer. The material has been applied to, and performs well on, several computational problems including function approximation, the travelling salesman problem and robot control [36,37]. However, the material has so far produced only modest performances on challenging RC benchmark tasks [9]. As a reservoir, the material has been shown to perform well on simple tasks, but struggles to exhibit strong nonlinearity and sufficient memory for more complex tasks [38,39].

In previous work [9,38,39], a small level of characterization was carried out on different CNT-based reservoirs, showing even the best fabricated material (a 1% concentration of CNTs w.r.t. weight mixed with poly-butyl-methacrylate) typically exhibits low MC, despite different biasing and stimulation methods for configuration.

The right concentration and arrangement of CNTs, and method for stimulating and observing the material, is still an open question. So far, the methods and materials used have led to overall modest performances on benchmark tasks such as NARMA-10 [9] and the Santa Fe laser time-series prediction task [38], but encouraging when the number of inputs and outputs are taken into account.

Characterizing a black-box material like the CNT composite is challenging because of its disordered structure and stochastic fabrication process, making it impractical (or even impossible for the general case) to model its exact internal workings. Originally, the CNT–polymer composite was proposed as a sandpit material to discover whether computer-controlled evolution could exploit a rich partially constrained source of physical complexity to solve computational problems [40]. Because of its physicality, with somewhat unknown computing properties, it provides a challenging substrate for the CHARC framework to characterize. Further details about the CNT-based substrate and its parameters are provided in appendix Cb.

### (c) Quality of test substrates

**Figure 5.**
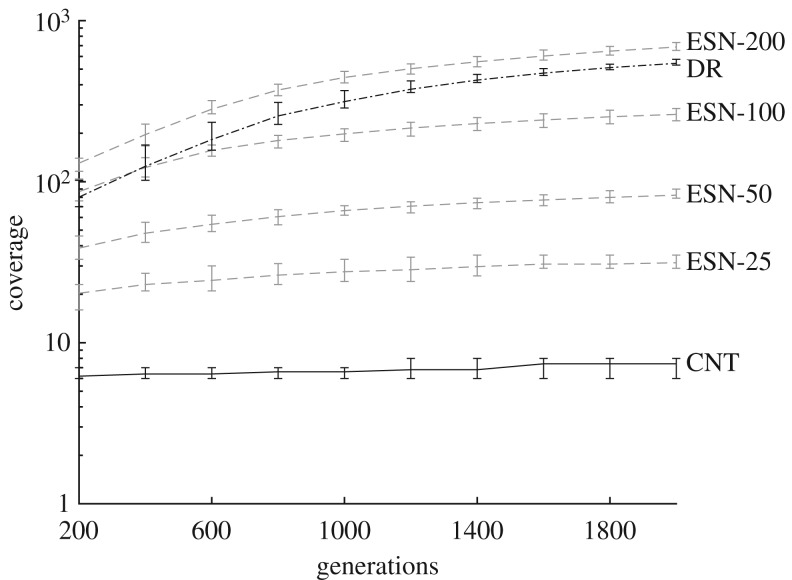
Voxel measure of coverage as number of generations increase. Test substrates are shown as black lines, reference substrates are dashed grey lines. Error bars display the min-max values for all search runs. Note the logarithmic coverage scale.

A visualization of exploration level *P*2.2 and the results of the evaluation level *P*2.3 for each substrate are presented here. Similar to phase one, the quality of each substrate is calculated as the total number of voxels occupied after 2000 search generations. Figure 5 shows the total number of occupied voxels after every 200 generations, with error bars displaying the min–max values for different evolutionary runs.

The differences in behavioural freedom between the DR, CNT and ESN substrates are significant. Using the voxel measure, we can determine which of the reference substrates are close equivalents in terms of quality to the test substrates. At the beginning of the search process, the DR appears similar in quality to an ESN with 100 nodes, while the CNT has a considerably smaller quality than the ESN of 25 nodes. As the number of search generations increases, the DR's coverage increases rapidly, reaching a final value close to an ESN with 200 nodes, yet the CNT struggles to increase its coverage. The rate at which behaviours are discovered for the DR and CNT are very telling, suggesting it is much harder to discover new behaviours for the CNT than the DR. This increased difficulty could imply the bounds of the substrate's behaviour space have almost been met: as the discovery rate of new novel behaviours decreases, either the search is stuck exploiting a niche area, or it has reached the boundaries of the whole search space.

**Figure 6.**
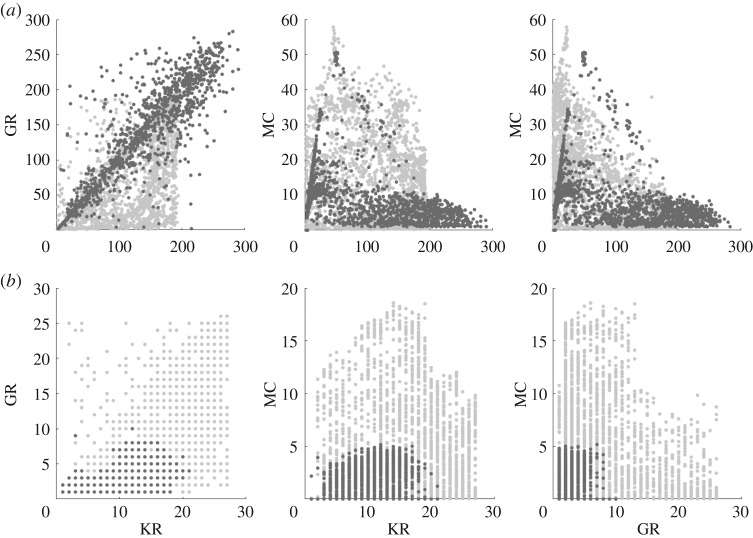
Behaviours discovered when exploring the ESN, CNT and DR substrates. To visually compare substrates, each test substrate is plotted over the reference substrate with the most similar quality. (*a*) 200 node ESN (light grey) with DR (dark grey), (*b*) 25 node ESN (light grey) with CNT (dark grey).

A visual inspection of the covered behaviour spaces provides a more detailed understanding of the final quality values. The discovered behaviours for both substrates are shown in figure 6. In each subplot, the behaviours for each test substrate (DR in figure 6*a* and CNT in figure 6*b*) are presented in the foreground and reference substrates with the most similar quality (200 node ESN in figure 6*a* and 25 node ESN in figure 6*b*) are placed in the background.

In figure 6*a*, the DR behaviours extend into regions that the 200 node ESN cannot reach, and, as a consequence, only sparsely occupies regions occupied by the ESN. Given more search generations, these sparse regions would likely be filled, as similar behaviours are already discovered.

The DR struggles to exceed the MC of the 200 node ESNs, or exhibit a KR or GR beyond 300, despite having 400 virtual nodes. This could indicate that increasing the number of virtual nodes does not necessarily lead to greater memory or dynamical variation, a feature more typical of ESNs (figure 11*d*). However, the virtual network size is not an isolated parameter; the time-scale and nonlinearity of the single node, and the separation between virtual nodes, all play an important role in reservoir dynamics.

In figure 6*b*, the CNT exploration process struggles to find behaviours with MC > 5, reaching what appears to be an MC limit. The highest discovered KR and GR values are also small, tending to be lower than (almost half) their possible maximum values, i.e. the total number of electrodes used as outputs. This suggests the substrate struggles to exhibit enough (stable) internal activity to create a strong nonlinear projection, and to effectively store recent input and state information, agreeing with previous results [9,38,39]. The results here also highlight why only a limited range of tasks are suitable for the substrate, and why small ESNs tend to be good models of the substrate.

These results show the CNT substrate in its current form features a limited set of behaviour, explaining its usefulness to only a small class of problems. The DR system features greater dynamical freedom, implying it can perform well across a larger set of problems. The coverage of this particular Mackey–Glass system is similar to large ESNs, explaining why they can closely match the performance of ESNs across the same class of problems [4,41].

### (d) Prediction transfer

The final level here, *P*2.4, evaluates the substrate-independence of the framework. To do this, we evaluate the transferability of the learnt relationships (FFNNs) from level *P*1.4 by measuring their prediction accuracy when tasked to predict a different substrate. We evaluate how well the trained models (FFNNs) of the reference substrates predict the performance of the other reference substrates, i.e. predict the task performance of different ESN sizes.

**Figure 7.**
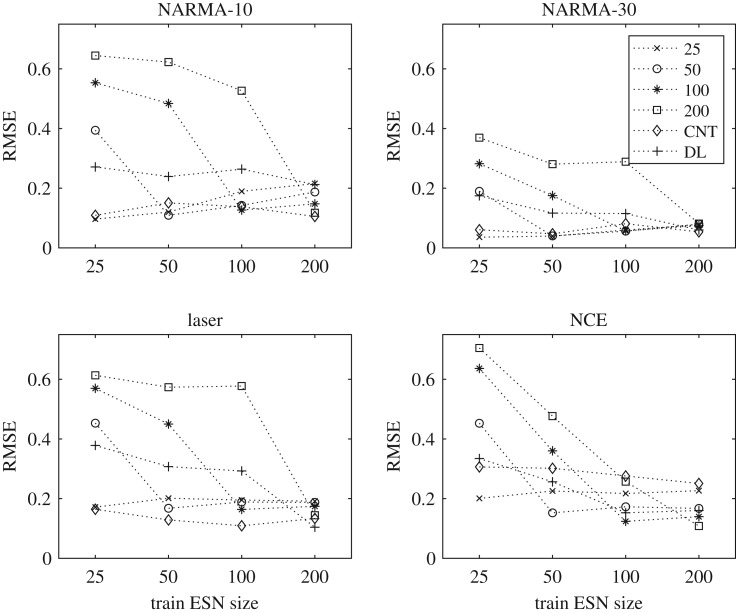
Prediction error (RMSE) of the learned models (FFNNs) from *P*1.3 when set to predict the task performance of other substrates. The modelled reference substrate (i.e. ESN size) used for the prediction is given on the *x*-axis and the test substrate is given in the legend.

Figure 7 shows the mean prediction error (RMSE) of all FFNNs for every predicted substrate. Each dashed line represents the predicted substrate. The *x*-axis represents the FFNNs trained on different reference network sizes; four sizes are shown for each task, being FFNNs trained using the ESN sizes 25, 50, 100 and 200 nodes. The *y*-axis is the prediction error (RMSE) of each model for each substrate.

The results show that the models trained with smaller network sizes tend to poorly predict the task performance of larger networks across all tasks. This intuitively makes sense; the smaller network models are trained without any data examples beyond their own behavioural limits, and thus cannot make an accurate prediction for larger networks.

The models trained with larger network sizes tend to predict the smaller networks fairly well. The best predictions occur when the model is trained and tested using the same network size. Considering the variation in task performance as size increases, and fewer training examples within specific areas occupied by smaller network sizes, prediction appears to be reasonably robust when using the largest explored reference substrate.

The model of the largest network (200 node) tends to better predict the DR, on average resulting in the lowest prediction errors. For the CNT, models of all network sizes result in low prediction errors for most tasks, except the nonlinear channel equalization task. Prediction error for this task, however, continues to improve as network size increases. Given these results, we argue that a reference substrate with high quality will tend to provide a good prediction of lower quality substrates.

**Figure 8.**
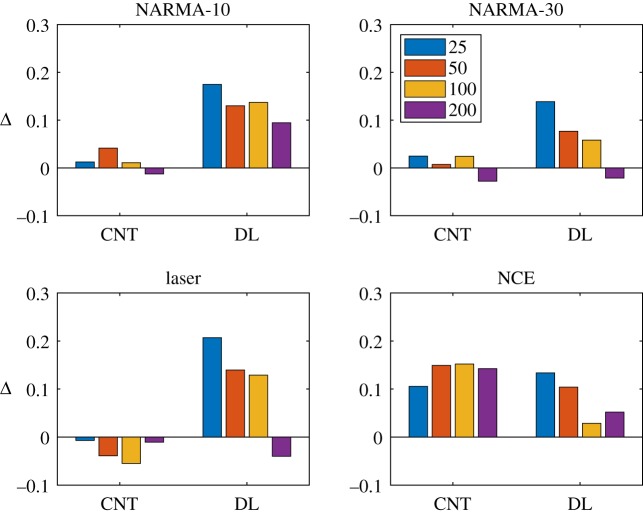
Difference (Δ) between best (self-)prediction and test prediction for CNT and DR substrates.

**Figure 11.**
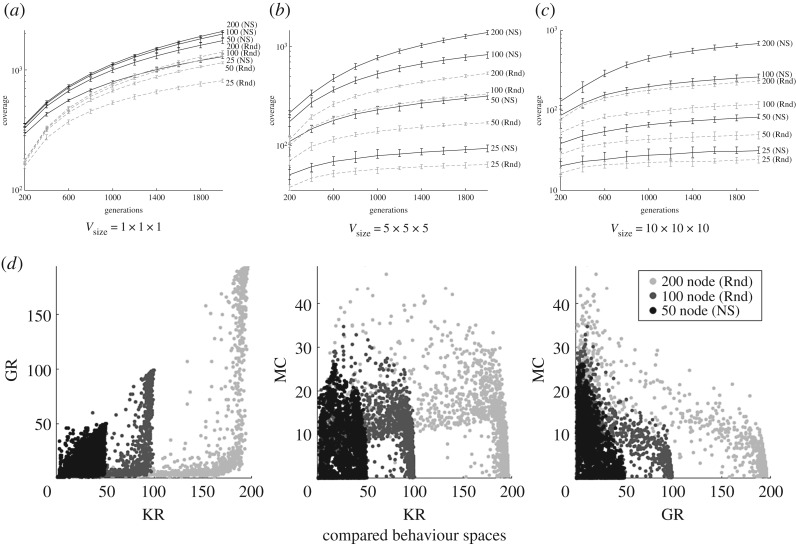
Average coverage (over 10 runs) of behaviour space against number of generations. Four network sizes and the two search methods are shown: NS (black, solid line) and random search (grey, dashed line). Error bars show minimum and maximum coverage. Quality is given for all network sizes, for both random and NS. (*a*) *V*_size_ = 1 × 1 × 1, (*b*) *V*_size_ = 5 × 5 × 5, (*c*) *V*_size_ = 10 × 10 × 10, (*d*) Compared behaviour spaces.

Figure 8 summarizes the results of the substrate-independence experiment. It plots the difference (Δ) between the best prediction error and the test substrates prediction error. When the overall prediction error is low and the difference (Δ) is close to zero, the relationship between behaviour and task performance is strong, and thus the abstract behaviour space reliably represents underlying computational properties, independent of the substrate's implementation.

Figure 8 plots Δ for the two test substrates on all four benchmark tasks. Each bar signifies the difference Δ between the best prediction error (from the model trained and tested with the same network size) and the trained model used to predict the test substrate. The results show on average the CNT tends to provide the smallest Δ with models of smaller networks. For the DR, the model of the largest network tends to provide Δ's closest to zero.

Overall, the low and similar prediction errors across substrates indicates that the CHARC framework has a good level of substrate independence. The results also highlight the non-trivial nature of modelling the task–property relationship, with some tasks being more difficult to model and predict than others. Although not the original purpose of this level, this demonstrates that one could roughly predict the task performances of newly characterized substrates, or potentially even test new tasks using a trained model without having to evaluate the test substrate directly. This feature of the framework is potentially beneficial to hardware systems where training can be time and resource intensive.

## 5. Conclusion

A fundamental question in RC is: For a given task, what characteristics of a dynamical system or substrate are crucial for information processing? The CHARC framework tackles this question by focusing on the characteristic behaviour of the substrate rather than its performance on a specific task. In the process, two non-trivial problems were attempted: (i) how to characterize the quality of a substrate for RC and (ii) how do computational properties relate to performance.

To use the framework, two phases must be completed. In the first phase, the basic levels (definition, exploration and evaluation) are applied to a reference substrate, providing context for future quality characterizations for other substrates. In the second phase, the test substrate is explored, characterized and compared.

The presented framework is flexible, allowing new computational measures, techniques and additional high-level functions to be added. In this work, we have proposed and demonstrated just one possible high-level function that could model the challenging relationships between tasks and computational properties. This is used to predict the task performance of the substrate given its task-independent behaviour.

Using the framework, we have shown that exploration through open-ended evolution can be a powerful tool for outlining the limitations and capability of a substrate. This explains why a CNT-based composite can solve some simple computational tasks but often struggles to compete with more complex reservoir substrates. It is also shown why DR compare so favourably to ESNs due to similar behavioural quality.

The characterization process of CHARC has many potential future applications, for example assessing the effect structure, topology and complexity has on dynamical freedom; using quality to guide, understand and explore substrate design; and, eventually, the design of suitable computational models. Ultimately, this can open the door for the co-design of both computational model and substrate to build better, more efficient unconventional computers.

## Figures and Tables

**Figure 9.**
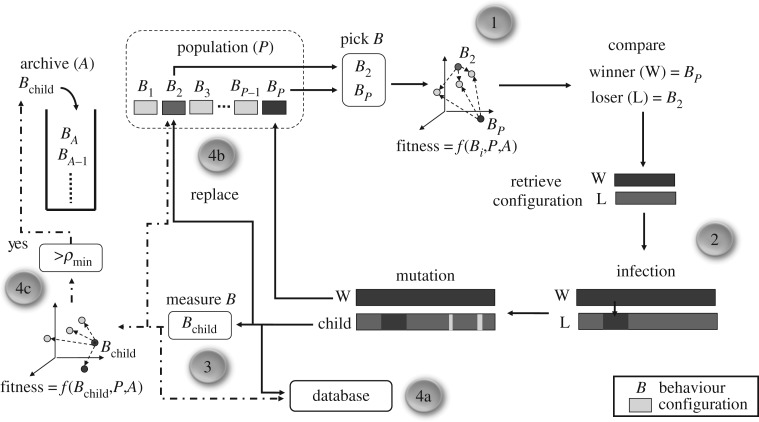
Adapted microbial GA with NS.

**Figure 10.**
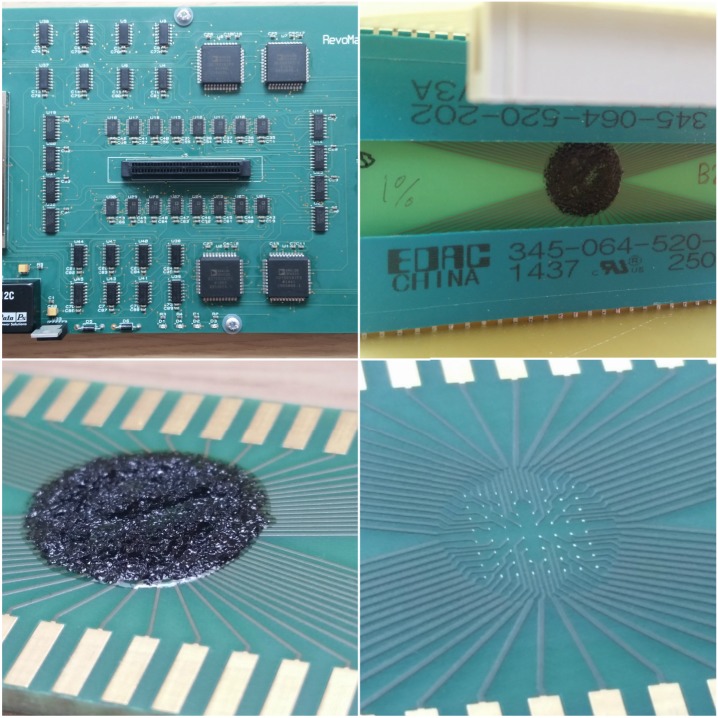
Hardware reservoir system. Micro-electrode housing, routing switch board and CNT–polymer deposited onto PCB electrode array. (Online version in colour.)

**Figure 12.**
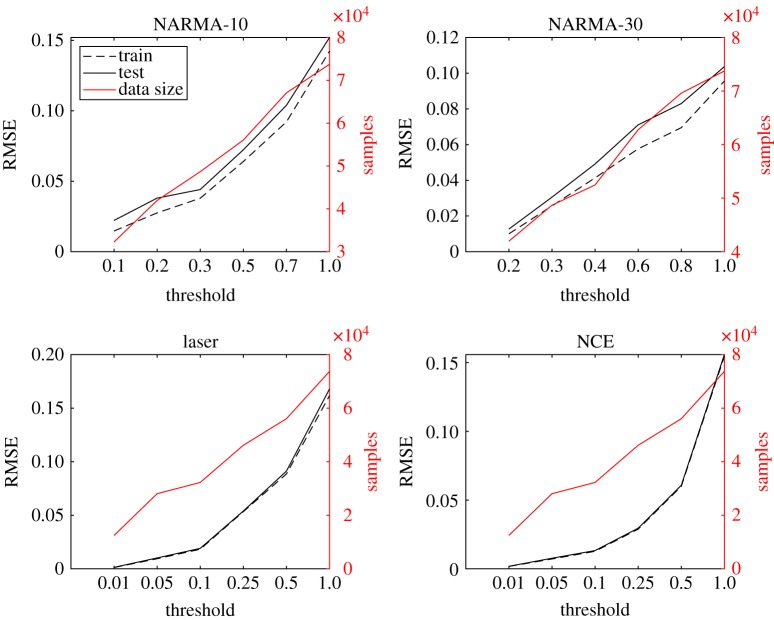
Test accuracy (RMSE) of the FNN when trained and tested on task performances below a threshold. The number of behaviours producing task performances below the threshold is also given. (Online version in colour.)

**Figure 13.**
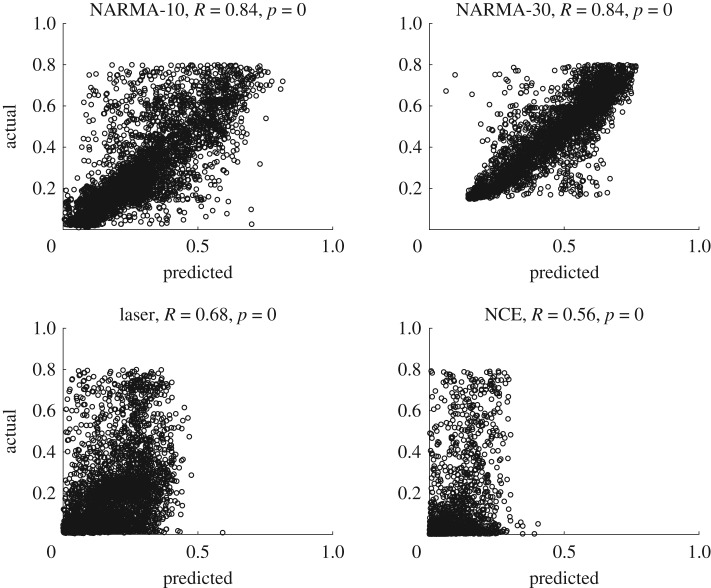
Plots of predicted performance versus actual performance of different behaviours, on different tasks. In each plot, approximately 7000 task performances (NMSEs) are shown of behaviours discovered using a 200-node ESN. *R* is Spearman's *ρ* correlation coefficient: all correlations are significant at the 95% confidence level.

## Footnotes

1 The term ‘substrate’ is used here to refer to any physical or virtual system that realizes a reservoir computer: any dynamical system featuring configurable parameters and a method to observe system states.

## Data accessibility

The research material (source code and data) can be accessed at github.com/MaterialMan/CHARC-Framework.git.

## Appendix A. Novelty search

In the presented framework, an open-ended evolutionary algorithm called NS [23–25] is used. NS is used to characterize the substrate's behaviour space, i.e. the dynamical freedom of the substrate, by sampling its range of dynamical behaviours. In contrast to objective-based techniques, a search guided by novelty has no explicit task-objective other than to maximize novelty. NS directly rewards divergence from prior behaviours, instead of rewarding progress towards some objective goal.

NS explores the behaviour space by promoting configurations that exhibit novel behaviours. Novelty of any individual is computed with respect to its distance from others in the behaviour space. To track novel solutions, an *archive* is created holding previously explored behaviours. Contrary to objective-based searches, novelty takes into account the set of all behaviours previously encountered, not only the current population. This enables the search to keep track of (and map) lineages and niches that have been previously explored.

To promote further exploration, the archive is dynamically updated with respect to two parameters, *ρ*_min_ and an update interval. The *ρ*_min_ parameter defines a minimum threshold of novelty that has to be exceeded to enter the archive. The update interval is the frequency at which *ρ*_min_ is updated. Initially, *ρ*_min_ should be low, and then raised or lowered if too many or too few individuals are added to the archive in an update interval. Typically in other implementations, a small random chance of any individual being added to the archive is also set.

In the presented implementation, a small initial *ρ*_min_ is selected relative to the behaviour space being explored and updated after a few hundred generations. *ρ*_min_ is dynamically raised by 20% if more than 10 individuals are added and *ρ*_min_ is lowered by 5% if no new individuals are added; these values were guided by the literature [23].

To maximize novelty, a selection pressure rewards individuals occupying sparsely populated regions in the behaviour space. To measure local sparsity, the average distance between an individual and its *k*-nearest neighbours is used. A region that is densely populated results in a small value of the average distance, and in a sparse region, a larger value. The sparseness *ρ* at point *x* is given by

A 1 $$\rho (x)=\frac{1}{k}\sum_{i=1}^{k}\mathrm{dist}(x,\xi_{i}),$$

where *ξ*_*i*_ are the *k*-nearest neighbours of *x*.

The search process is guided by the archive contents and the current behaviours in the population, but the archive does not provide a complete picture of all the behaviours explored. Throughout the search, the population tends to meander around existing behaviours until a new novel solution exceeding the novelty threshold is discovered. To take advantage of this local search, all the explored behaviours are stored in a separate database *D*. This database stores all the information used to characterize the substrate's later quality and has no influence on the search, which uses only the archive.

**(a) Novelty search implementation**

In the literature, NS is frequently combined with the Neural Evolution of Augmented Topologies (NEAT) [25,42] representation; this neuro-evolutionary method focuses on adapting network topology and complexifying a definable structure. For the CHARC framework, a more generic implementation is needed, featuring a minimalistic implementation not based on any specific structure or representation. For this reason, an adaptation of the steady-state Microbial Genetic Algorithm (MGA) [43] combined with NS is used. The MGA is a genetic algorithm reduced to its basics, featuring horizontal gene transfer (through bacterial conjugation) and asynchronous changes in population where individuals can survive long periods.

To apply the MGA to the problem a number of adaptations are required. Caching fitness values in the standard steady-state fashion is not possible, as fitness is relative to other solutions found and stored in the growing archive. In this implementation, no individual fitnesses are stored across generations; however, the same steady-state population dynamics are kept, i.e. individuals are not culled, and may persist across many generations.

An overview of the evolutionary loop is given in figure 9. The complete process is also outlined in pseudo-code in algorithm 1.

At the beginning of the search process, a random population is created. In the population, both the substrate configurations and the resulting behaviours *B* are stored. This initial population is then added to the archive *A* and database *D*.

At step 1, tournament selection with a tournament size of two is used. To ensure speciation, the first parent is picked at random and the second is chosen within some proximity to the other determined by the MGA parameter *deme size*. In this step, the fitness values (novelty) of both behaviours are calculated relative to population *P* and archive *A*. The individual with the larger distance, that is occupying the less dense region of the behaviour space, is adjudged the winner. This elicits the selection pressure towards novel solutions. The microbial GA differs from other conventional GAs as the weaker (here, less novel) individual becomes ‘infected’ by the stronger (more novel) one, replacing its original self in the population.

At step 2, the configurations of both behaviours are retrieved and manipulated. This constitutes the infection and mutation phase. In the infection phase, the weaker parent undergoes horizontal gene transfer becoming a percentage of the winner and loser. The genetic information of the weaker parent does not disappear in this process, as some percentage defined by the recombination rate parameter remains intact. In the mutation phase, the weaker parent undergoes multiple point-mutations, becoming the new offspring.

At step 3, the configuration of the new offspring is untested, therefore the behaviour *B*_Child_ of the individual needs to be updated. At steps 4*a* and 4*b*, the offspring's behaviour and configuration are added to the database *D* and it replaces the loser in the population *P*.

At the last step 4*c*, the fitness/novelty of the offspring *B*_Child_ is compared to both the current population *P* and archive *A*. If the novelty of the offspring exceeds the novelty threshold *ρ*_min_, the behaviour *B*_Child_ (configuration is not needed) is added to the archive *A*.

Overall, three fitness values are calculated at each generation. Two fitness evaluations occur in the selection phase and a third fitness evaluation is carried out on the offspring, in order to update the archive. The computational complexity of the fitness function is *O*(*nd* + *kn*) using an exhaustive *k*-nearest neighbour search. As the dimension *d* of the archive/behaviour space is small (*d* = 3 property measures), the number of *k*-neighbours (here *k* = 15) has the dominant effect. This value of *k* is chosen experimentally; larger *k*-values improve accuracy but increase run time. As the archive size increases, run time increases proportional to archive size *n*. To reduce complexity, Lehman & Stanley [25] describe a method to bound the archive using a limited stack size. They find that removing the earliest explored behaviours, which may result in some limited backtracking, often results in minimal loss to exploration performance.

The same NS parameters are applied to every substrate. These are generations limited to 2000; *population* *size* = 200; *deme* = 40; *recombination* *rate* = 0.5; *mutation* *rate* = 0.1; *ρ*_min_ = 3 and *ρ*_min_ *update* = 200 generations. Five evolutionary runs are conducted for the CNT and delay-based reservoir, as the time to train increases significantly, and 10 runs for the ESN substrates.

## Appendix B. Benchmark tasks for prediction phase

The NARMA task evaluates a reservoir's ability to model an *n*th order highly nonlinear dynamical system where the system state depends on the driving input and state history. The task contains both nonlinearity and long-term dependencies created by the *n*th-order time-lag. An *n*th ordered NARMA task predicts the output *y*(*n* + 1) given by equation (B 1) when supplied with *u*(*n*) from a uniform distribution of interval [0, 0.5]. For the 10th-order system parameters are: *α* = 0.3, *β* = 0.05, *δ* = 0.1 and *γ* = 1; for the 30th-order system: *α* = 0.2, *β* = 0.004, *δ* = 0.001 and *γ* = 1.

B 1 $$y(t+1)=\gamma \left(\alpha y(t)+\beta y(t)\left(\sum_{i=0}^{n-1}y(t-i)\right)+1.5u(t-9)u(t)+\delta \right).$$

The laser time-series prediction task predicts the next value of the Santa Fe time-series Competition Data (dataset A).^2^ The dataset holds original source data recorded from a Far-Infrared-Laser in a chaotic state.

The nonlinear Channel Equalization task introduced in [45] has benchmarked both simulated and physical reservoir systems [31]. The task reconstructs the original i.i.d signal *d*(*n*) of a noisy nonlinear wireless communication channel, given the output *u*(*n*) of the channel. To construct reservoir input *u*(*n*) (see equation (B 3)), *d*(*n*) is randomly generated from − 3,  − 1,  + 1,  + 3 and placed through equation (B 2):

B 2 $$\begin{array}{rl} q(n) & =0.08d(n+2)-0.12d(n+1)+d(n)\\ & \;+0.18d(n-1)-0.1d(n-2)\\ & \;+0.091d(n-3)-0.05d(n-4)\\ & \;0.04d(n-5)+0.03d(n-6)+0.01d(n-7) \end{array}$$

B 3 $$\begin{array}{rl} u(n) & =q(n)+0.036q{\left(n\right)}^{2}-0.011q{\left(n\right)}^{3}. \end{array}$$

Following [45], the input *u*(*n*) signal is shifted +30 and the desired task output is *d*(*t* − 2).

## Appendix C. Substrate parameters

**(a) Echo state networks**

In phase one, regardless of network size the same restrictions are placed on global parameter ranges and weights, applying the same weight initiation processes each time. For example, global parameters ranges include an internal weight matrix (*W*) scaling between [0, 2], scaling of the input weight matrix (*W*_in_) between [−1, 1], and the sparseness of *W* [0, 1]. For both random and NS, at creation, a reservoir has each global parameter drawn from a uniform random distribution, as well as input weights and internal weights drawn uniformly from other ranges; *W*_in_ between [−1, 1] and *W* between [−0.5, 0.5].

**(b) Carbon nanotube–polymer**

The training and evaluation of the carbon-based substrate is conducted on a digital computer. Inputs and representative reservoir states are supplied as voltage signals. The adaptable parameters for evolution are the number of input–outputs, input signal gain (equivalent to input weights), a set of static configuration voltages (values and location) and location of any ground connections. Configuration voltages act as local or global biases, perturbing the substrate into a dynamical state that conditions the task input signal.

In this work, a 1% CNT poly-butyl-methacrylate (CNT/PBMA) mixture substrate is investigated. The substrate was mixed and drop cast onto a micro-electrode array using the same process in [9,38,39]. The electrode array comprises 64 electrodes (contact sizes of 100 μm and spacings of 600 μm between contacts) deposited onto a FR-4 PCB using a chemical process that places nickel and then a layer of gold (figure 10).

Two National Instruments DAQ cards perform measurements and output analogue voltages; a PCI-6225 (16-Bit, 250 KS/s, with 80 analogue inputs), and PCI-6723 (13-Bit, 800 KS/s, with 32 analogue outputs). Both cards communicate to a desktop PC through a session-based interface in Matlab. The PCI-6723 supplies an additional 8 digital I/O lines to a custom routing board to programme on-board switches and synchronize the cards.

**(c) Delay-based reservoir**

To generate *N* virtual nodes and collapse them into a usable state observation, time-multiplexing is used. The input signal *u*(*t*) is sampled and held for the period *τ* (the length of the delay line) and mixed with a random binary mask *M*, perturbing the node away from the relaxed steady state. For an interval defined by the node separation *θ* = *τ*/*N*, the mask is applied as a piecewise constant, forming the input to the nonlinear node *J*(*t*) = *I*(*t*)**M*. The state of the *i*th virtual node is obtained after every *τ*, as: *x*_*i*_(*t*) = *x*(*τ* − (*N* − *i*)*θ*).

The model of the Mackey–Glass dynamical system is described as

C 1 $$\begin{array}{r} \dot{X}(t)=-X(t)+\frac{\eta \cdot [X(t-\tau )+\gamma \cdot J(t)]}{1+{\left[X(t-\tau )+\gamma \cdot J(t)\right]}^{p}}, \end{array}$$

where *X* represents the state, $\dot{X}$ its derivative with respect to time, and *τ* is the delay of the feedback loop. The parameters *η* and *γ* are the feedback strength and input scaling. The exponent *p* controls the nonlinearity of the node. The parameter *T*, typically omitted from equation (C 1), represents the characteristic time-scale of the nonlinear node. In order to couple, the virtual nodes and create the network structure, *T*≥*θ* is required. Together, all these parameters determine the dynamical regime the system operates within.

The parameters of the delay-based reservoir in this work are fixed at *T* = 1, *θ* = 0.2, *τ* = 80 and *N* = 400, based on values given in [4]. During the exploration process, evolution can alter the mask, flipping between the binary values [−0.1, 0.1] and manipulate all of the Mackey–Glass parameters: 0 < *η* < 1, 0 < *γ* < 1 and the exponent 0 < *p* < 20.

## Appendix D. Property measures

**(a) Kernel and generalization rank**

The kernel measure is performed by computing the rank *r* of an *n* × *m* matrix *M*, outlined in [20]. To create the matrix *M*, *m* distinct input streams *u*_*i*_, …, *u*_*m*_ are supplied to the reservoir, resulting in *n* reservoir states *x*_*u*_*i*__. Place the states *x*_*u*_*i*__ in each column of the matrix *M* and repeat *m* times. The rank *r* of *M* is computed using singular value decomposition (SVD) and is equal to the number of non-zero diagonal entries in the unitary matrix. The maximum value of *r* is always equal to the smallest dimension of *M*. To calculate the effective rank, and better capture the information content, remove small singular values using some high threshold value. To produce an accurate measure of KR *m* should be sufficiently large, as accuracy will tend to increase with *m* until it eventually converges.

The GR is a measure of the reservoir's capability to generalize given similar input streams. It is calculated using the same rank measure as kernel quality, however each input stream *u*_*i*+1_, …, *u*_*m*_ is a noisy version of the original *u*_*i*_. A low generalization rank symbolizes a robust ability to map similar inputs to similar reservoir states.

**(b) Memory capacity**

A simple measure for the linear short-term MC of a reservoir was first outlined in [21] to quantify the *echo state* property. For the echo state property to hold, the dynamics of the input-driven reservoir must asymptotically wash out any information resulting from initial conditions. This property therefore implies a fading memory exists, characterized by the short-term memory capacity.

To evaluate memory capacity of an *N* node reservoir, we measure how many delayed versions *k* of the input *u* the outputs *y* can recall, or recover with precision. Memory capacity MC is measured by how much variance of the delayed input *u*(*t* − *k*) is recovered at *y*_*k*_(*t*), summed over all delays.

D 1 $$\begin{array}{r} \mathrm{MC}=\sum_{k=1}^{2N}{\mathrm{MC}}_{k}=\sum_{k=1}^{2N}\frac{{\mathrm{cov}}^{2}(u(t-k),y_{k}(t))}{\sigma^{2}(u(t))\sigma^{2}(y_{k}(t))}. \end{array}$$

A typical input consists of *t* samples randomly chosen from a uniform distribution between [0 1]. Jaeger [21] demonstrates that ESNs driven by an i.i.d. signal can possess only *MC* ≤ *N*.

A full understanding of a reservoir's memory capacity cannot be encapsulated through a linear measure alone, as a reservoir will possess some nonlinear capacity. Other memory capacity measures proposed in the literature quantify the nonlinear, quadratic and cross-memory capacities of reservoirs [3].

## Appendix E. Effect of voxel size

To evaluate quality, a simple voxel based measure is used.

The coverages of the different search methods and network sizes using the minimal voxel size are given in figure 11*a*. Here we see that small voxel sizes significantly overestimate the dynamical freedom/quality of networks explored using NS. Smaller networks such as the 25 and 50 node ESNs are seen to occupy similar or more voxels than larger networks, suggesting similar or better quality. However, when visually comparing each explored behaviour space (figure 11*d*) we see the measure fails to account for diversity and spread of behaviours. This demonstrates the importance of selecting an appropriate voxel size, as a voxel size too small cannot differentiate between local areas that are highly populated, and fewer behaviours spread across a greater distance.

To reduce the problem, the voxel size must be increased. By how much depends on the size of the behaviour spaces being compared. As a guide, when comparing drastically different systems, a larger voxel size will tend to differentiate better. Of course, a voxel size too big will also struggle to differentiate between systems. Because of this potential biasing problem, a visual inspection of the behaviour space is always recommended. Examples of different voxel sizes are given in figure 11.

## Appendix F. Reliability of behaviour space representation

To train the FFNNs in §3d, the Levenberg–Marquardt algorithm [46] was used for 1000 epochs, with training dataset as 70% of the database *D*, and 30% set aside for testing. To gather statistics, 10 FFNNs were trained and tested for each network size and task.

Determining the reliability of the behaviour space representation is challenging. Selecting suitable data for this modelling process is difficult as some behaviours perform particularly poorly on tasks, reducing the overall prediction accuracy of the model. Poor task performing reservoirs tend to increase noise in the models training data as some appear to be randomly scattered across the behaviour space.

To reduce the problem, different thresholds were placed on the training data to show how well the relationship of the highest performing reservoirs can be modelled. Applying each threshold, reservoirs with task performance (NMSE) above the threshold are removed from the training and test data.

A low prediction error (RMSE) of the model with low thresholds indicates greater ability to predict high performing reservoirs. At higher thresholds, more training data are available but include reservoirs that perform poorly on the task.

The mean prediction errors of 10 feed-forward networks, trained with each threshold, on each task, using the behaviours from the 200 node ESNs, are shown in figure 12. Across all tasks, the accuracy of the model improves when smaller thresholds are used, i.e. error is smallest when predicting only the highest performing reservoirs, suggesting a strong relationship between behaviour space and task performance.

To visualize how well the relationship is modelled for task performances of NMSE < 1, we plot the predicted NMSE versus the evaluated NMSE in figure 13. Here, the output of four FFNNs, trained for each task, are given. We see the laser and nonlinear channel equalization tasks are harder to model, typically resulting in an overestimation, as the actual task performances of most behaviours tend to be low, generally with an NMSE < 0.2. We also calculate Spearman's *ρ* (called *R* here), a non-parametric test measuring the strength of association between predicted and actual. A value of 1 indicates perfect negative correlation, while a value of +1 indicates perfect positive correlation. A value of 0 indicates no correlation between predicted and actual. The *p*-value for each measure is also provided. If the *p*-value is less than the significance level of 0.05, it indicates a rejection of the null hypothesis that no correlation exists, at the 95% confidence level. The high values of *R* and essentially zero *p*-values suggest the models predict task performance very well.
